# Alternative scheduling of pulsatile, high dose sunitinib efficiently suppresses tumor growth

**DOI:** 10.1186/s13046-016-0411-2

**Published:** 2016-09-07

**Authors:** Maria Rovithi, Richard R. de Haas, Richard J. Honeywell, Dennis Poel, Godefridus J. Peters, Arjan W. Griffioen, Henk M. W. Verheul

**Affiliations:** Department of Medical Oncology, VU University Medical Center, Amsterdam, The Netherlands

**Keywords:** Cancer, Sunitinib, Pulsatile, High dose, Alternative scheduling, CAM model, Apoptosis, Autophagy, Tumor growth

## Abstract

**Background:**

Increased exposure to multitargeted kinase inhibitor sunitinib is associated with improved outcome, emphasizing the importance of maintaining adequate dosing and drug levels. The currently approved schedule (50 mg daily, four weeks on, two weeks off) precludes further dose-intensification. Recent data suggest that sunitinib, although initially developed as an antiangiogenic agent, has direct antitumor activity.

**Methods:**

In this study, we tested whether a chemotherapy-like schedule of pulsatile high dose sunitinib would result in improved antitumor activity.

**Results:**

In vitro, a single exposure to 20 μM sunitinib for 6-9 h resulted in complete inhibition of tumor cell growth and cell death conveyed through activation of caspases and autophagy upregulation. Notably, repeated exposure of tumor cells to pulses of high concentrations of sunitinib did not induce resistance. In vivo, once-weekly treatment with high dose sunitinib of tumors growing on the chorioallantoic membrane (CAM) of the chicken embryo significantly impaired tumor growth by 57 % compared to vehicle, outperforming the daily, standard scheduling.

**Conclusions:**

These results prompted the initiation of a phase I clinical trial, where intermittent, high dose sunitinib is being investigated in patients with advanced solid tumors (registration number and date: NCT02058901, 30 September 2013, respectively). The trial is actively recruiting patients and promising preliminary indications of antitumor activity have been observed.

## Background

Competitive inhibition of aberrantly or mutationally activated protein kinases downregulates critical hallmarks of cancer growth [1]. The introduction of tyrosine kinase inhibitors (TKIs) elicited remarkable therapeutic responses in malignancies previously regarded as chemoresistant [2]. Despite these important strides, the field of TKIs faces significant challenges, spanning from low response rates and lack of selectivity to development of drug resistance [3]. A crucial and challenging aspect in the clinical development of TKIs is to determine a biologically active dose as well as an optimal treatment schedule [4–6].

Sunitinib malate, (SUTENT; Pfizer, New York, NY), an orally administered TKI, targets multiple receptors, including the vascular endothelial growth factor receptor (VEGFR) and platelet-derived growth factor receptor (PDGFR). It has already been approved for the treatment of advanced renal cell cancer (RCC), gastrointestinal stromal tumors (GIST) and pancreatic neuroendocrine tumors (pNET). The currently approved dose is 50 mg daily for 4 weeks followed by a 2-week off period (4/2 schedule) [4, 7, 8]. Various dose schedules have been assessed in order to improve the efficacy of sunitinib. Daily and every-other-day sunitinib administrations have been explored which incorporated planned rest periods for both recovery from toxicities and concerns of drug accumulation with continuous dosing [9–11]. The relationship between sunitinib exposure, efficacy and safety has been evaluated in a recent pharmacokinetic/pharmacodynamic (PK/PD) meta-analysis. An exposure-response model demonstrated that increased area under the curve levels of sunitinib resulted in greater efficacy [12]. A recent study also demonstrated that continuous daily dosing of sunitinib at the lower dose of 37.5 mg has no benefit in efficacy and safety over the standard 4/2 schedule [13]. In addition, although sunitinib was developed as an antiangiogenic agent, the drug has been shown to exert direct anti-tumor activity [14].

We recently demonstrated in patients that tumor concentrations of sunitinib are significantly higher compared to their corresponding plasma concentrations (averaging on 9.5 μM vs 0.3 μM, respectively). At this tumor concentration, sunitinib was shown to inhibit proliferation of various tumor cell lines in a dose-dependent manner, including renal-, breast- and colon cancer cells. At high concentrations, complete blockade of proliferation and even induction of tumor cell death was observed [15]. In this study, we evaluated whether pulsatile exposure of cells to high concentrations of sunitinib would induce cell death and whether application of this scheduling would suppress tumor growth in vivo.

## Methods

### Cell culture

The tumor cell lines 786-O, HT-29, MDA-MB-231, H1650 and A431 were cultured in Dulbecco's Modified Eagle's Medium supplemented with 5 % FBS and maintained in a humidified incubator containing 5 % CO_2_ at 37 °C. All cell lines were authenticated by BaseClear, Leiden, Netherlands by STR profiling (last date of authentication: June 2014). Sunitinib was provided by Pfizer Global Pharmaceuticals and was prepared as 20 mmol/l stock solution in dimethyl sulfoxide (DMSO; Sigma-Aldrich) and stored at -20 °C.

### Proliferation assays

Cytotoxicity of sunitinib was assessed by MTT assay as described previously [15]. Briefly, cells were seeded in a 96-well plates and allowed to adhere for 24 h. A *t* = 0 measurement was carried out and cells were treated with increased concentrations of sunitinib for the indicated time intervals, followed by washing and maintaining in DMEM medium (5 % FBS) for a total of 144 h. Next, 100 μl of MTT indicator dye (5 mg/ml) was added to each well and the cells were incubated for 2 h at 37 °C. After addition of 100 μl of DMSO solution to each well, absorption was measured at 540 nm in a microplate reader (Spectra Fluor Tecan, Salzburg, Austria). The reading taken from the wells with cells cultured in control medium was used as 100 % viability value. Cell proliferation was calculated using the following formula: % of proliferation = [(144 h measurement of treated cells − 0 h measurement)/(144 h measurement of untreated cells − 0 h measurement)] × 100 %. Subtracting the measurement at the beginning of treatment (*t* = 0 measurement) might result in negative value, representing cell killing [14]. All experiments were performed in triplicate and repeated at least three times.

### Intracellular measurement of sunitinib concentration

Utilizing the fluorescent properties of sunitinib, we produced initially a concentration dilution series of sunitinib (0.01 to 40 μM) in medium only, that was measured in microplate reader (Spectra Fluor Tecan, Salzburg, Austria), to build a concentration curve. Consecutively, 5,000 cells per well were plated in 96-well plate, allowed to attach overnight and exposed to the indicated sunitinib concentrations for 2 h. Medium was removed; cells were washed with PBS and lysed in M-PER Mammalian Protein Extraction Reagent supplemented with protease and phosphatase inhibitor cocktails (Pierce). Fluorescence was measured again, the value of the blank well (containing cells but no drug) was subtracted and after correlation with the concentration curve and correction for the number of cells, the intracellular concentration of sunitinib was calculated.

### Cell cycle and cell death analysis

Cells (150,000 cells /well) were seeded in 6-well plates. After drug exposure as indicated, cells were trypsinized, resuspended in medium collected from the matching samples and centrifuged at 1,200 rpm for 5 min. Cells were stained with propidium iodide buffer (0/1 mg/ml, 0.1 % RNAse) on ice in the dark. Subsequently, DNA content was analyzed with FACSCalibur flowcytometer (Becton-Dickinson, Immunocytometry Systems, San Jose, USA). Extent of cell death was calculated by the subG1 peak.

### Western blot analysis

After treatment as indicated, cells were lysed in Mammalian Protein Extraction Reagent (M-PER) supplemented with protease and phosphatase inhibitor cocktails (Pierce). Micro BCA protein assay (Pierce) was used for determination of protein concentrations. Samples containing 50 μg protein underwent electrophoresis on 8 % to 12 % SDS polyacrylamide gels and were subsequently transferred to PVDF membranes. Proteins were detected using the following antibodies: LC3B (#2775, Cell Signaling), SQTM1/p62 (#8025, Cell Signaling) and β-actin (A5441; Sigma-Aldrich). After incubation with IRDye (infrared dye)-labeled secondary antibodies (LI-COR Biosciences), membranes were scanned and analyzed with the Odyssey Infrared Imaging System and accompanying software program (LI-COR Biosciences; [16].

### Determination of caspases 3/7 activity

786-O cells were plated in 96-well plate (5,000 cells/well) and after overnight incubation, were treated with the indicated concentrations for the indicated time intervals. Activity of caspases 3/7 was determined using Apo-ONE® Homogeneous Caspase-3/7 Assay, according to the manufacturer’s protocol (http://worldwide.promega.com/resources/protocols/technical-bulletins/0/apoone-homogeneous-caspase-3-7-assay-protocol/).

### CAM assay

Fertilized chicken White Leghorn eggs were incubated in a hatching incubator with a relative air humidity of 65 % and a temperature of 37 °C. On post-fertilization day 6, CAM surface was gently scratched and 5 x 10^6^ HT29 cells in suspension with 50 % matrigel to a total volume of 50 μl were grafted on the CAM. Sunitinib 40 mg/kg, a dose extrapolated from mice experiments, was applied daily topically on the tumors on Embryonic Developmental Day (EDD) 12-20 for the daily schedule while sunitinib 120 mg/kg was applied topically on the tumors only twice, on days 12 and 20 for the weekly schedule. Dose for both schedules was calculated based on the weight of the chicken embryo at the start of treatment (EDD12). Tumor volume was calculated using an external caliper and by the modified ellipsoid formula ½ x (length x width^2^). Plasma and tumors were collected on EDD21. Tumors were weighed and cut, one half was snap frozen and the other half was fixed in zinc-fixative solution and embedded in paraffin.

### Sunitinib measurements in CAM samples

Concentrations of sunitinib in the plasma and tumor samples collected from the CAM was determined with liquid chromatography—tandem mass spectrometry (LC/MS-MS). Following weighing and overnight freeze-drying of tumors, 200 μl of ice-cold 83 % acetonitrile (ACN) was added for extraction and ultrasonicated for 10 min. After centrifugation, 50 μl of supernatant was transferred for LC/MS-MS analysis as previously reported for plasma and cell pellet homogenates [17].

### Immunohistochemistry

Immunohistochemical staining was performed on 5 μm thick paraffin sections of CAM tumors. Following deparaffinization in xylene and rehydration through a graded series of alcohol, endogenous peroxidase activity was blocked by 20 min incubation in 0.3 % H_2_O_2_/PBS. Next, antigen retrieval was performed in sodium citrate solution (pH 6.0) using a pressure cooker. After blocking in 5 % BSA/PBS for 30 min at room temperature (RT), the samples were incubated for 1 h at RT with the primary antibody diluted in 0.5 % BSA/PBS. Following primary antibodies were used: CD31 (SZ31, Dianova) and Ki-67 (M7240; Dako; 1:50). Control slides were incubated with 0.5 % BSA/PBS. Next, the slides were incubated for 30 min at RT with the appropriate secondary biotinylated antibody, followed by incubation with strep-ABC-HRP for 30 min at RT (1 μl avadin and 1 μl biotin in 500 μl PBS). Finally, staining was visualized with 3,3-diamino-benzidine-tetra hydrochloride (DAB), 0.3 mg/ml in 1 ml PBS with 0.3 % H_2_O_2_. All slides were counterstained with haematoxylin and mounted in Entellan (Merck) for microscopy. Three areas with the greatest amount of neovascularization from each tumor section were selected and the microvessels in these areas were counted under 40× objective lens. Any brown-stained endothelial cell or endothelial cell cluster that could be clearly separated from the adjacent structures, with or without a vessel lumen, was considered as a single, countable microvessel. The numbers of microvessels in the three areas were averaged to give mean microvascular density (MVD). Cancer proliferation of CAM tumors was calculated as the ratio of the number of nuclei stained for Ki67 to the total number of nuclei per field and expressed as the percentage of Ki67-positive nuclei. Minimum of three pictures of each slide were taken at 10x magnification and quantification of positive DAB staining was done using Image J with colour deconvolution [18].

### Statistical analysis

Results were subjected to statistical analysis using GraphPad Prism v4.0 software. One-way ANOVA was followed by Student's *t*-test, two-tailed and unpaired. Data are expressed as means ± SEM or SD when appropriate. Grubbs’ test was applied for exclusion of significant outliers (*p* < 0.05 at significance level of a = 0.05). A *p* value less than 0.05 was considered to be statistically significant.

## Results

### Pulsatile, high dose sunitinib inhibits tumor cell proliferation

**Fig. 1:**
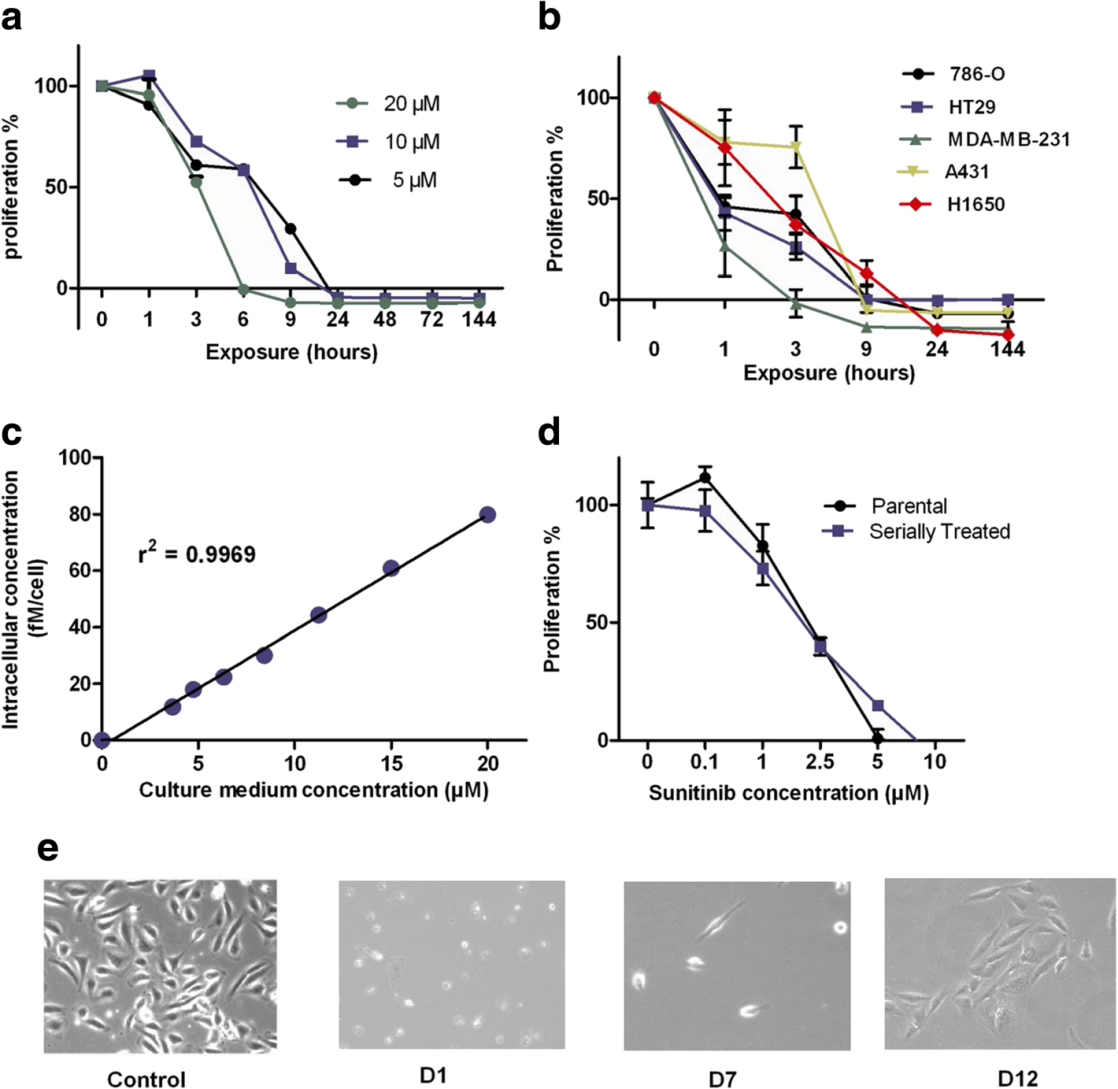
Short exposure to high concentrations of sunitinib provokes tumor cell death, while serially treatment with this scheduling does not induce resistance. **a** 786-O RCC cells were exposed to 5, 10 and 20 μM of sunitinib for the depicted time intervals, ranging from 1 – 144 h exposure. Proliferation was studied at 144 h with MTT. **b** The indicated cancer cell lines were exposed to 20 μM of sunitinib for the various time intervals (range 1- 144 h). Proliferation was studied at t = 144 h with MTT. Exposure to 20 μM of sunitinib for 9 h resulted in tumor cell death, independent of cell line. **c** 786-O RCC cells were exposed to indicated sunitinib concentrations (range 0 – 20uM) for 6 h and intracellular accumulation of sunitinib was calculated. **d** Sensitivity to sunitinib, determined with proliferation assay, of cells sequentially treated with 20 μM for 9 h (*n* = 4 times) was compared to the sensitivity of untreated cells. **e** Photos depicting the regrowth of 786-O RCC cells after 9 h exposure to 20 μM of sunitinib. Control, untreated, D, days after exposure. Error bars, SEM

The 786-O renal cancer cell line was exposed to various concentrations of sunitinib (5, 10 and 20 μM) for time intervals ranging from 1 h to 1 week. In all cases, cell viability was determined using the MTT assay, at the end of the experiment after 7 days. We show that sunitinib in this pulsatile scheduling was a potent inhibitor of cell proliferation in this scheduling. Exposure to 20 μM of sunitinib for 1 to 3 h decreased cell proliferation by 50 % while incubation for 6 to 9 h resulted in complete inhibition of the tumor cell viability (Fig. 1a). Exposure concentration inversely correlated to exposure time, as similar inhibition of cell proliferation at lower concentrations was reached only after prolonged exposure (continuous incubation with 5 μM for 24 h, Fig. 1a). Similar results were obtained with cell lines of different tumor types (HT-29, H1650, MDA-MB231, A431), to exclude a cell line specific effect (Fig. 1b). The cellular uptake of sunitinib was linear across the concentration range (Fig. 1c).

### Pulsatile exposure to high dose sunitinib does not induce resistance

We evaluated whether serial pulse application of high doses would induce resistance to sunitinib. 786-O cells were treated for 9 h with 20 μM of sunitinib after which fresh medium was applied and cells were kept in culture to allow regrowth. When these cultures reached confluency after approximately two weeks, the same sunitinib schedule was applied. Sensitivity to sunitinib of these repetitively treated cells (*n* = 4 times) was tested in comparison to untreated cells. Both cell types were equally sensitive to sunitinib with IC_50_ values of 2 μM. (Fig. 1d, e).

### Reduced cell viability by pulsatile sunitinib is mediated by apoptosis

**Fig. 2:**
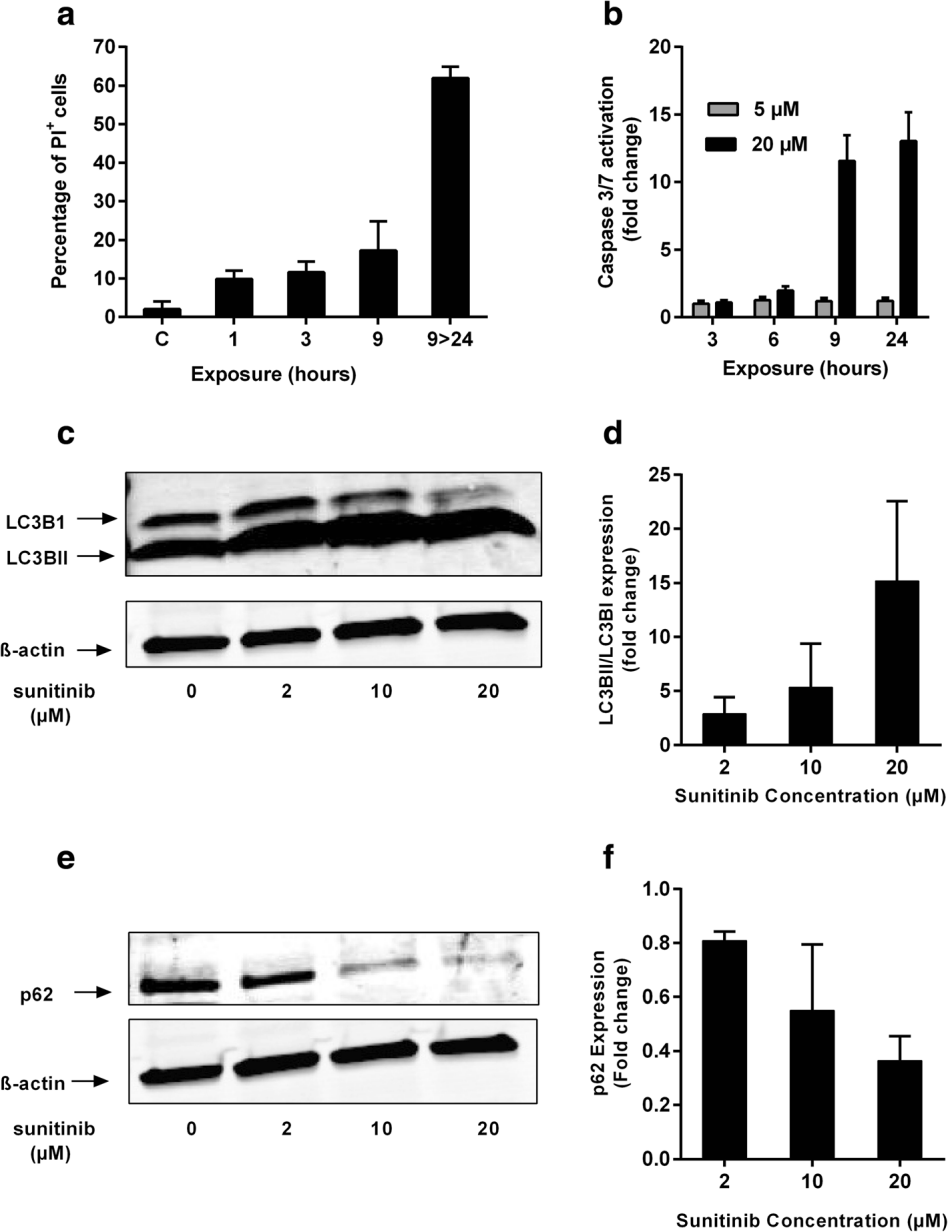
Pulsatile, high concentration of sunitinib leads to increase in the sub-G1 population, activation of caspase-3/7 and upregulation of autophagic flux. **a** FACS analysis utilizing PI staining of 786-O RCC cells exposed to 20 μM of sunitinib after 1, 3, 9 h or 15 h after the 9-h exposure (9 > 24). Sunitinib leads to proportional increase in sub-G1 population, indicative of cell death. **b** 786-O RCC cells were exposed for 3, 6, 9 and 24 h to 5 or 20 μM of sunitinib and activation of caspase-3/7 was measured. Data presented relative to control = 1. **c**, **e** LC3BI, LC3BII and p62 protein levels in control and treated with the indicated concentrations of sunitinib cell lysates were analyzed by Western blotting. **d**, **f** Quantification of LC3BII/LC3BI conversion ratio and p62 expression, respectively. For both figures, protein level was initially normalized towards the loading control, β-actin, and then towards the corresponding control- untreated sample

It has already been reported that, independent of angiogenesis inhibition, sunitinib exerts direct antitumor effects [14]. As shown in Fig. 2a, the percentage of PI positive cells, arrested at the subG1 cycle phase, increased to 17 % after 9 h-exposure to 20 μM sunitinib in 786-O RCC cells. This percentage increased further to 61.5 % when these cells were subsequently washed and incubated for 24 h in drug-free medium (Fig. 2a). To further examine the contribution of apoptosis to the mesured cell death, we determined the activation of executioner caspase-3/7 after exposure of 786-O cells to 5 and 20 μM of sunitinib. We observed a concentration-dependent increase in activation of caspase-3/7. Whereas short or prolonged exposure to 5 μM of sunitinib failed to activate caspase-3/7, exposure to 20 μM of sunitinib for 9 h resulted in a 12-fold increase in caspase-3/7 activation. Comparable induction of caspase activation was observed only after longer (24 h) exposure (13- fold increase, Fig. 2b).

### Induction of autophagic flux after short exposure to high concentrations of sunitinib

In cell lysates from 786-O cells exposed to increasing concentrations of sunitinib (2, 10 and 20 μM) for 6 h, protein levels of microtubule-associated protein 1 light chain 3 I and II (LC3BI, LC3BII, respectively) and p62 were determined by western blotting to indicate the impact of treatment on the autophagic flux. Six hours exposure to 2, 10 and 20 μM of sunitinib induced a concentration-dependent increase in the conversion ratio of LC3BII/LC3BI of 2.9, 5.3 and 15.2- fold, respectively, compared to baseline expression of control cells (Fig. 2c and d). A simultaneous decrease in the levels of p62 was observed (20 %, 45 % and 64 % decrease compared to control, after exposure to 2, 10 and 20 μM of sunitinib, respectively, Fig. 2e and f). This increase in the conversion ratio, in combination with the increased degradation of p62, denotes a relevant increase in the autophagic flux, possibly as a stress response to sunitinib exposure.

### Pulsatile, high dose sunitinib exhibits in vivo antitumor activity and results in high intratumoral drug concentration

The CAM model can efficiently support the growth of tumor cells, thereby offering a reliable way to study primary tumor angiogenesis, anti-tumor therapies and underlying molecular pathways in a biologically relevant system that is both cost and time-effective. It can adequately bridge the gap between monolayer cell cultures and the intricate murine models, providing insights into tumor growth and drug efficacy in an expedited way, since tumors already form only 4 days after cell inoculation. Simultaneously, high dose treatment with sunitinib is not feasible in an in vivo murine tumor model. Previously maximally double or triple the established dose (40 to 60 mg/kg) has been used as high dose while solubility issues preclude further dose increase [19]. Therefore, we investigated whether pulsatile, high dose treatment with sunitinib would have antitumor activity on HT29 xenograft tumors grown on the chorioallantoic membrane of the chicken embryo (CAM). Limitation of this in vivo model is the relatively short length of time due to the grow of the embryo, which prohibits further testing after 16 days of tumor inoculation [20].

**Fig. 3:**
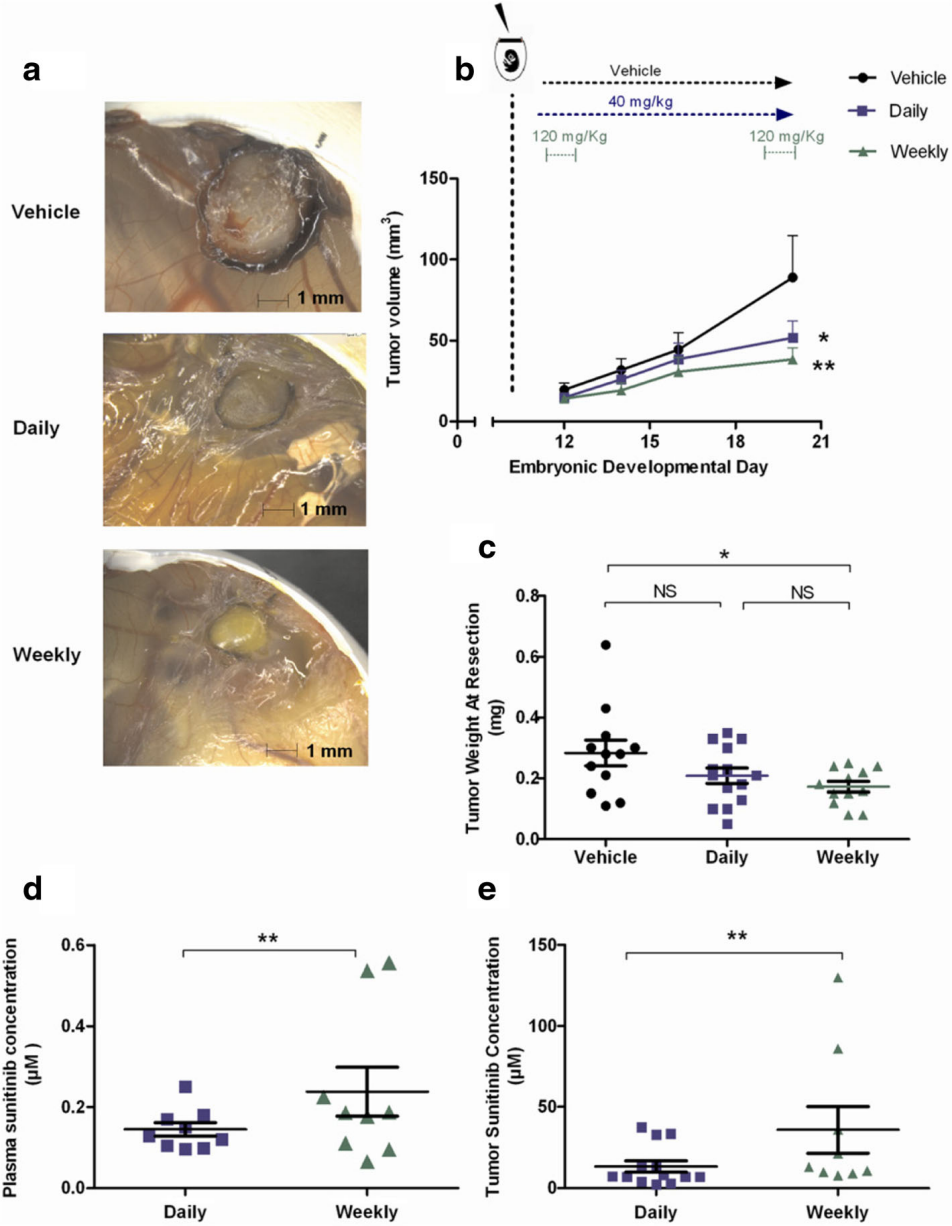
Pulsatile, high dose of sunitinib in vivo results in inhibition of tumor growth and significantly higher plasma and intratumoral sunitinib concentrations compared to the standard, daily schedule. **a** Photos of HT29 tumors growing on the CAM representative of the three treatment arms (vehicle, 40 mg/kg of sunitinib daily from EDD12 to EDD20 (designated as “daily) or 120 mg/kg twice per week, on EDD12 and EDD20 (designated as “weekly”). **b** Increase in tumor volume during treatment for the three treatment arms. Error bars, SEM. **c** Weight (in mg) of the collected tumors, in each treatment arm. **d**, **e** Concentration of sunitinib in the plasma and the collected tumors per treatment arm. Error bars, SD.*, *p* < 0.05, ** *p* < 0.01, NS: not significant

**Fig. 4:**
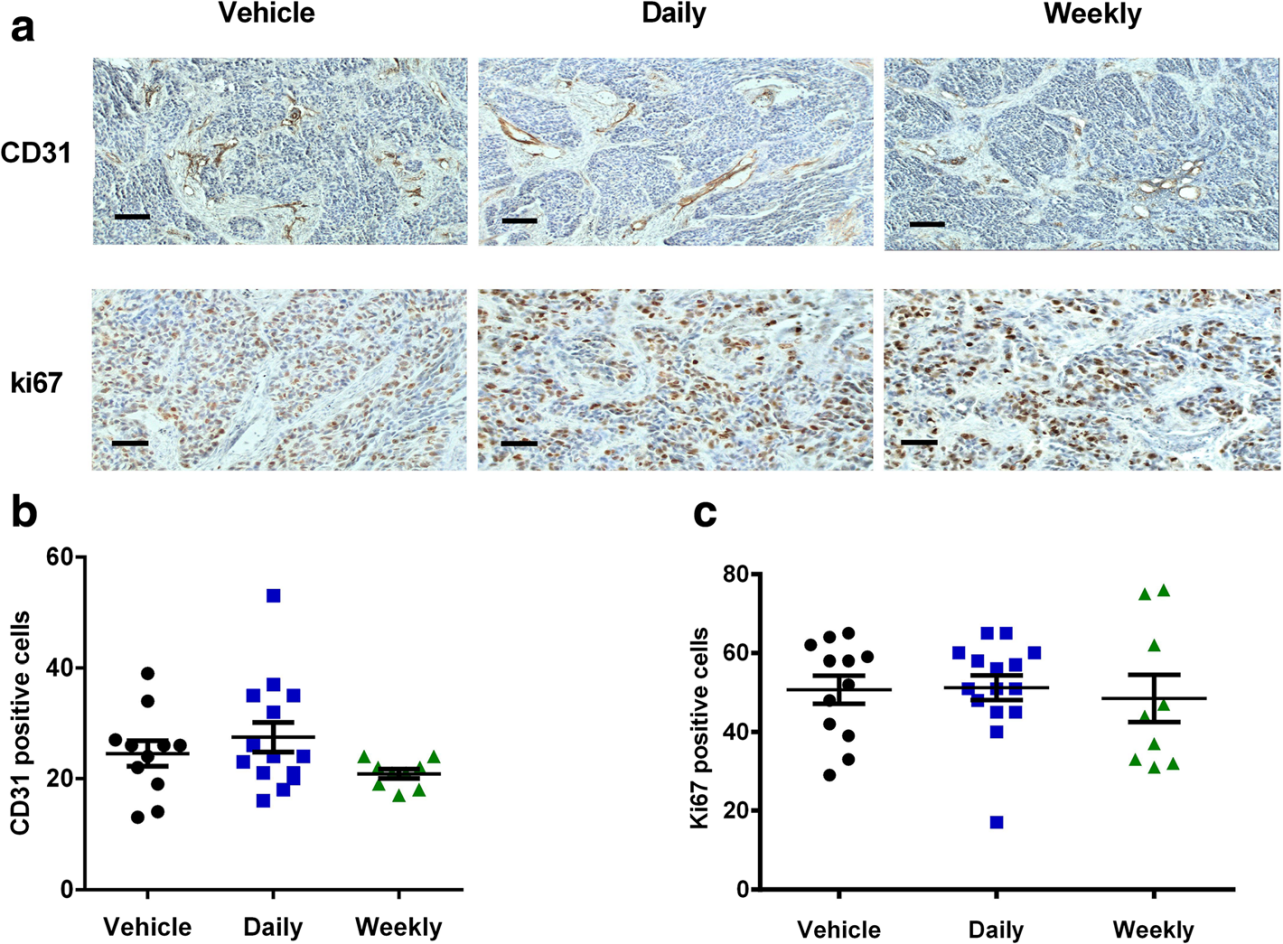
Pulsatile, high dose sunitinib has no effect on tumor microvessel density (MVD) or on proliferation rate of cancer cells. **a** Representative photos of (immunohistochemical) staining of tumors established on the CAM from HT-29 tumor cells, treated as indicated. Upper panel: CD31 staining; lower panel: Ki-67 staining **b** Quantification of microvessel density (MVD) using CD31 staining. Data are expressed as number of CD31 positive cells per field, x10 magnification. Scale bars, 100 μm **c** Quantification of tumor cell proliferation using Ki-67 staining. Data are expressed as percentage of Ki-67 positive tumor cells to total tumor cells per field, 40x magnification. Scale bars, 50 μm.Error bars, SD

Daily application of the commonly in vivo used sunitinib dose (40 mg/kg) indeed induced a significant delay in tumor growth (41 % decrease compared to vehicle treated group, *p* < 0.05). However, this effect was even more prominent in the treatment group of once weekly, high dose sunitinib of 120 mg/kg, that resulted in a 57 % decrease in tumor growth compared to vehicle (*p* < 0.01) (Fig. 3a, b). This inhibitory activity was confirmed on the tumor weight after resection (mean tumor weight 0.17 mg (range: 0.08-0.25 mg) vs 0.23 mg (range: 0.05-0.56 mg) vs 0.33 mg (range: 0.11-0.87 mg) for the weekly vs daily treatment vs vehicle group respectively; one way ANOVA *p*:0.018 and Student’s *t*-test for the daily vs weekly: *p* = 0.02 (Fig. 3c). This weekly, pulsatile high dose treatment led also to higher plasma sunitinib concentrations compared to the daily schedule (0.23 μM (range: 0.09-0.537 μM) vs 0.18 μM (range: 0.09-0.25 μM), respectively, *p* = 0.0014), and higher tumor concentrations (36 μM (range: 7.8-130 μM) for the weekly vs 13.5 μM (range 7.0-37.4 μM) for the daily schedule, *p* = 0.0002) (Fig. 3d, e). The observed growth inhibition after pulsatile high dose scheduling seems to occur irrespective of angiogenesis inhibition, as denoted by the lack of inhibitory effect on tumor microvessel density (MVD; number of CD31+ vessels per 40x field: 24 (range: 13-39) vs 27 (range: 16-53) vs 21 (range 17-24) for the vehicle vs daily sunitinib vs weekly sunitinib group, respectively) (Fig. 4a, b). Simultaneously, no differences in the Ki67 expression were found among the control, the daily and the weekly schedule (Fig. 4a, c).

## Discussion

The drug development of sunitinib is based on the concept that inhibition of angiogenesis function induces tumor cell death indirectly, through substrate deprivation. Disassociating sunitinib from the concept of antiangiogenesis- only mediating function, we hypothesized that an alternative dose scheduling could potentiate its efficacy against a variety of cancer types. Our results show that pulsatile high dose sunitinib can potently inhibit tumor growth both in vitro and in vivo.

Short exposure to high concentrations of sunitinib in vitro inhibited cancer cell proliferation as efficiently as prolonged exposure to lower concentrations, without inducing resistance. Apoptosis was involved in the cytocidal process while induction of autophagy potentially indicates a prosurvival reaction of the cells after exposure to sunitinib. Consequently, intermittent application of high doses of sunitinib on tumors growing on the CAM in vivo model resulted in increased plasma and intratumoral drug concentrations and significant inhibition of tumor growth.

Optimizing TKI treatment regimens and dosing is an important research field, aiming at improved antitumor efficacy with acceptable toxicity and avoidance of induction of resistance. It is debatable whether the currently approved TKI treatment scheduling is optimal in terms of achieving maximum therapeutic responses. Challenging the notion that prolonged protein kinase inhibition is required to translate into efficacy, Shah et al. reported that transient potent BCR-ABL inhibition by dasatinib induces apoptotic pathways in chronic myeloid leukemia cells [21]. Weekly, 10-fold higher than the currently approved, doses of erlotinib have been reported as salvage therapy in patients with non-small cell lung cancer and leptomeningeal metastases, with an acceptable toxicity profile [22]. Interestingly, in 83 patients with metastatic melanoma who received treatment with daily sorafenib and PK-guided dose escalation, high area under the curve (AUC) was the only parameter in the multivariate analysis that correlated to efficacy [23]. Based on their observation of markedly reduced sorafenib exposure at disease progression compared to baseline, Arrondeau et al. proposed dose escalation aiming at restoration of drug exposure and thereby treatment efficacy [24].

For imatinib, significantly lower plasma levels have been correlated to a lack of response in patients with chronic myeloid leukemia (CML) [25] and GIST [26]. Results from the phase II 1046 axitinib trial [27], suggested that dose titration resulting in higher exposure is associated with improved efficacy [28]. Chein et al., translating data from mouse models where lapatinib in intermittent, high doses demonstrated improved efficacy compared to the standard continuous low-dose therapy, reported recently a phase I trial investigating this schedule in patients with advanced solid tumors [29]. Though an apparent exposure ceiling seemed to preclude further dose escalation in the current formulation, high-dose, intermittent lapatinib was well tolerated and resulted in significantly increased plasma concentrations. Additionally, a relationship between lapatinib exposure and biologic activity was established; patients with plasma concentrations approximating 10,000 ng/ml presented marked responses, while all patients with low lapatinib plasma concentrations exhibited progressive disease [29].

For sunitinib it was recently found that continuous administration of 37.5 mg sunitinib failed to exhibit superior activity versus the approved four weeks on and two weeks off treatment schedule of 50 mg sunitinib daily. These findings not only further supported current clinical practice, but also hinted that off drug periods do not seem to compromise efficacy [12]. Since cumulative evidence point to the relation of exposure to efficacy in this class of agents, serial PK measurements are needed to identify patients with suboptimal exposure [30]. Employment of our pulsatile high dose sunitinib treatment resulted in antitumor activity without induction of resistance. This result could have been anticipated, since in vitro resistance induction in cell lines requires prolonged exposure to these agents [31]. Interestingly, it was recently demonstrated that discontinuous scheduling of vemurafenib preempts the appearance of drug resistance, due to the regression of drug-dependent, already established drug-resistant clones in the interfering drug-free periods [32].

In our study, we report that sunitinib induces caspase-dependent cell death. Previously, the antitumor activity of sunitinib has been ascribed to activation of apoptosis in tumor or endothelial cells and it has furthermore been correlated to the baseline expression level of its target-encoding genes [33]. In addition, exposure of cancer cells to this scheduling of sunitinib upregulates autophagy, probably in the context of a pro-survival process and in line with previous reports that autophagy acts as an adaptive mechanism responding to, among others, antiangiogenic treatment [34]. Whether the upregulation of autophagy is a prosurvival reaction of cells or a cell death pathway that is induced by this type of treatment scheduling remains an open question, which requires further exploration. We did not test separately the effect of the active metabolite of sunitinib, N-desethyl sunitinib, but it is known that it exhibits similar potency compared to sunitinib in biochemical and cellular assays (Sutent, Summary of Product Characteristics, EMA).

Therefore, we would anticipate fully comparable results with the use of the metabolite.

The CAM model is able to efficiently support the growth of tumor cells, thereby offering a reliable way to study primary tumor formation and supportive angiogenesis [20]. Pulsatile scheduling in our in vivo model did not result into vessel pruning or tumor angiogenesis inhibition, both reported to provoke intratumoral hypoxia, an adaptive mechanism that has been implicated as a potential resistance mechanism [35]. One obvious explanation might be the short treatment window, a limiting factor in this model that may not allow perhaps sufficient time for development and detection of such antiangiogenic effects. Alternatively, inhibition of VEGF signaling in this context might ultimately affect functions distinct from angiogenesis, but implicated in tumorigenesis [36], such as the autocrine and paracrine VEGF signaling in tumor cells [36, 37]. However, we did not examine the potential antiangiogenic activity of treatment on the CAM membrane itself. Additionally, while sunitinib inhibited primary tumor growth, there was no effect on the cellular proliferation rate, as indicated by the stable percentage of ki-67 positive cells in all treatment arms; this might be again attributed to the limited treatment window but it could also fall in line with the recent observation of prolonged stability of tumor growth rate during sunitinib administration [38]. Our observed linear correlation between concentration and cellular uptake indicate that increased plasma concentrations might result in higher intratumoral concentrations. Data concerning the blood TKI levels that are required to achieve and maintain target inhibition are inadequate. For sunitinib, plasma concentrations above 50 ng/mL are considered potentially active [4]. In non-clinical tissue specimens, sunitinib concentration was found 13- to 308 fold higher than in plasma [39]. In our in vivo model, the intermittent application of a higher dose resulted in significantly higher plasma concentrations compared to the daily application of a lower dose, further leading to significantly higher intratumoral concentrations. The results for the daily schedule are directly comparable to previous reports on plasma and tumor concentrations of sunitinib in patients where sunitinib intratumoral concentrations are 14 to 30-fold higher than plasma concentrations, consistent with a large volume of distribution, reaching up to 50 μM after standard daily treatment [15, 40].

The potent efficacy of sunitinib has been postulated to result from simultaneous inhibition of individual target receptors both in cancer cells and in the supporting tumor vasculature [41]. Simultaneously, preclinical and early clinical data raised the concern that antiangiogenic treatment might potentiate the rate of metastases, accelerate tumor growth and lead to a compensatory angiogenesis boost; increase in cancer tissue hypoxia and pericyte ablation are proposed to mechanistically facilitate metastasis [19, 42, 43]. Contradictory data have been reported whereby a transient improvement in tumor oxygenation has been noted in tumor-bearing mice that receive treatment with sunitinib [44], while prevention of hypoxia correlates to metastasis inhibition [45]. This increase in tumor perfusion after anti-angiogenic treatment in patients has been correlated with improved prognosis [46], while selective eradication of pericytes was correlated to enhanced efficacy of antiangiogenic treatment [47]. Prospective clinical trials attending to the complex nature of these questions are lacking. However, recently Blagoev et al. addressed these concerns utilizing data from the pivotal phase III trial of sunitinib vs interferon-a in patients with RCC, that ultimately led to the approval of sunitinib as first line therapy in this indication. The authors concluded that no evidence indicated increased rate of tumor progression, following treatment with sunitinib, while pointing out the discrepancies with the preclinical data and commenting on the limitations of murine models [48]. Additionally, analysis of primary RCC tissues of patients preoperatively treated with sunitinib suggested that an interval longer of 2 weeks off drug is needed to observe increase in MVD that could facilitate tumor progression [43].

Our proposed pulsatile, high dose schedule might have a number of potential implications with respect to the clinical use of sunitinib. Since sunitinib presents with predictable, linear pharmacokinetics, one could assume that dose increases might lead to proportional increases in drug exposure, which in turn has been correlated to improved antitumor efficiency [12]. To further investigate our initial hypothesis and translating our preclinical data, we designed a phase I trial, where high dose sunitinib is given in an intermittent once weekly or once every two week schedule (ClinicalTrials.gov Identifier: NCT02058901). The primary aim of the study is the determination of Maximum Tolerated Dose (MTD) and the pharmacokinetic behavior of sunitinib, while an expansion cohort at the MTD level will refine dose optimization and may offer a preliminary assessment of the efficacy of this scheduling.

## Conclusions

Identification of the optimal dose and scheduling of kinase inhibitors, remains an open quest, ongoing even in the postapproval setting. We here show for sunitinib, that an increased exposure concentration requires a short exposure time to result into potent antitumor activity, both in vitro and in vivo. We have directly translated our findings in the context of a phase I trial. Implications of this trial could be the integration of this alternative scheduling of sunitinib in daily practice and generalized application of the same dosing strategy across small molecule development.

## Abbreviations

CAM: Chorioallantoic membrane of the chicken embryo
GIST: Gastrointestinal stromal tumor
LC3BI/LC3BII: Microtubule-associated protein 1 light chain 3 I and II respectively
PDGFR: Platelet-derived growth factor receptor
pNET: Pancreatic neuroendocrine tumor
RCC: Renal cell cancer
TKIs: Tyrosine kinase inhibitors
VEGFR: Vascular endothelial growth factor receptor
